# Quantifying hepatitis C transmission risk using a new weighted scoring system for the Blood-Borne Virus Transmission Risk Assessment Questionnaire (BBV-TRAQ): Applications for community-based HCV surveillance, education and prevention

**DOI:** 10.1186/1477-7517-5-12

**Published:** 2008-04-23

**Authors:** Mark A Stoové, Craig L Fry, Nicholas Lintzeris

**Affiliations:** 1 Research Fellow, Centre for Epidemiology and Population Health Research, Macfarlane Burnet Institute for Medical Research and Public Health, 85 Commercial Road, Melbourne, Victoria 3004, Australia; 2 Senior Research Fellow, Department of Health Science, Faculty of Medicine, Nursing and Health Sciences, Monash University, PO Box 527, Frankston, Victoria, 3199, Australia; 3 Senior Staff Specialist, Drug Health Services, Sydney South West Area Health Service, Page Building 5, Missenden Road, Camperdown, NSW 2050, Australia

## Abstract

**Background:**

The hepatitis C virus (HCV) is a major cause of drug-related morbidity and mortality, with incidence data implicating a wide range of HCV transmission risk practices. The Blood-Borne Virus Transmission Risk Assessment Questionnaire (BBV-TRAQ) is a content valid instrument that comprehensively assesses HCV risk practices. This study examines the properties of a new weighted BBV-TRAQ designed to quantify HCV transmission risk among injecting drug users (IDU).

**Methods:**

Analyses of cross-sectional surveys of Australian IDU (N = 450) were used to generate normative data and explore the properties of a weighted BBV-TRAQ. Items weights were assigned according to expert key informant ratings of HCV risk practices performed during the development stages of the BBV-TRAQ. A range of item weights was tested and psychometric properties explored. A weighting scheme was recommended based on the plausibility of normative subscale data in relation to research evidence and the ability of BBV-TRAQ scores to discriminate between HCV positive and negative participants.

**Results:**

While retaining the psychometric properties of the unweighted scale and demonstrating good internal reliability. By taking into account the *relative *transmission risk of a broad range of putative HCV practices, the weighted BBV-TRAQ produced promising predictive validity results among IDU based on self-report HCV status, particularly among young and less experienced injectors.

**Conclusion:**

Brief, easy to administer and score, and inexpensive to apply, the utility of the BBV-TRAQ for community based education and prevention is enhanced by the application of item weights, potentially offering a valid surrogate measure for HCV infection among IDU.

## Background

As a leading notifiable disease and a major cause of drug related morbidity and mortality, it is well recognised that the hepatitis C virus (HCV) poses a significant public health challenge. In addition to quality of life impairment at all disease stages [1,2], most individuals exposed to HCV become chronically infected (and infectious), with 10–20% developing cirrhosis or hepatocellular carcinoma [3].

In many countries the majority of HCV prevalence cases are comprised of current or former injecting drug users (IDU; [4-6]). Incidence data implicates a wider range of HCV transmission risk practices (e.g., environmental contamination) and injecting paraphernalia (e.g., spoon, filter, water, swab) other than the sharing of used syringes [7,8].

Such findings, in the context of continuing high prevalence of injecting risk behaviour [6,9-11] and HCV infection [12-14], suggest that controlling the spread of HCV is dependent on preventing transmission within the IDU population [8]. Although social and structural determinants of drug use and 'risk' are important to this end [15], HCV transmission is unlikely to be reduced without significant changes in the specific behaviours believed responsible for the spread of the virus [16]. Reductions in the prevalence of risk behaviours is therefore a necessary component of prevention responses and require an expansion of existing strategies through improved education and support for IDU [17].

One of the challenges in HCV surveillance and prevention rests with evaluating the efficacy of the broad range of interventions designed to reduce the prevalence of high-risk practices and, ultimately, rates of HCV seroconversion. To monitor HCV incidence, evaluate intervention efficacy or clarify the significance of putative/theoretical risk practices, longitudinal cohort studies represent a gold standard of evidence. These studies, however, are expensive to conduct and difficult to complete successfully in community-based samples (and therefore less likely to be funded in some jurisdictions) because high HCV prevalence and incidence rates in IDU necessitate the serial testing of very large numbers of participants. The capacity of current surveillance mechanisms are therefore impeded in terms of their capacity to evaluate prevention initiatives and identify HCV transmission risk practices that contribute most to new infections. This not only poses a significant barrier at the level of nationwide population prevention programs, but also for the multitude of local community and agency specific HCV prevention programs.

An alternative approach to sero-incident studies that is arguably better suited to local community level HCV prevention and evaluation is to assess participation in high-risk practices for HCV transmission. Until recently, this has been complicated by the lack of a standardised instrument capable of reliably measuring participation in a sufficiently broad range of injecting and other putative risk practices associated with HCV transmission. Although some injecting risk measures [18,19] demonstrate acceptable reliability and validity [20,21], they have poor content validity for HCV monitoring purposes due to insufficient coverage of the full range of HCV risk practices.

The predominant mode of HCV transmission is via risky injecting drug use practices [12,22]. Evidence also implicates tattoos playing a role in the spread of HCV, particularly those performed by non-professionals and/or in prison settings [23-26]. Sexual transmission of HCV is highly debated [27,28]. Although some methodologically suspect studies have suggested an 'appreciable' risk of sexually transmitted HCV [29], recent prospective cohort studies with considerable person-years of follow-up have concluded the risk to be extremely low and perhaps non-existent [30,31]. The risk of horizontal household transmission of HCV is also suggested to be similarly low [32,33].

The *Blood-Borne Virus Transmission Risk Assessment Questionnaire (BBV-TRAQ) *is a standardised content and construct valid instrument offering comprehensive assessment of injecting, sex and skin penetration risk practices for HCV, HBV, and HIV [34,35] and has been translated by the World Health Organisation into eight languages [36]. Although the comprehensive nature of the risk practices canvassed in the BBV-TRAQ is an advantage, each of these practices ultimately contribute equally to BBV-TRAQ scores, despite evidence suggesting they carry markedly different relative risks of transmitting a blood-borne virus (BBV).

This limitation makes it difficult for the current format BBV-TRAQ to offer a genuine and practical indication of the risk of contracting a BBV. This paper reports on the application of a weighting scheme for BBV-TRAQ items to take account of the relative risks associated with different risk practices. The analyses undertaken in this paper focus on HCV by providing initial normative data of HCV transmission risk among a sample of IDU, and explores the properties of the weighted scale to approximate overall HCV transmission risk in this group. IDU constitute the main HCV sero-prevalent and sero-incident risk group in many countries and constitute the population from which the BBV-TRAQ was originally developed. Thus this population was a logical choice from which to begin the iterative process of scale refinement described in this paper.

## Methods

### Design

Cross-sectional survey data of IDU was used to examine the properties of the weighted BBV-TRAQ. This data was collected between September 1999 and March 2000 as part of the Australian Blood-Borne Virus Risk and Injecting Drug Use Study (ABRIDUS; [37]). The aim of ABRIDUS was to measure the extent of specific HCV risk practices among IDU and explore the contextual determinants of these practices. The survey contained three sections: demographic information including history of drug use and self-report HIV, HCV, and HBV status; the BBV-TRAQ; and a semi-structured component investigating participants' usual injecting context in relation to injecting risk practices. The BBV-TRAQ was self-completed by participants in the presence of a research assistant, with other survey sections administered by research assistants. Ethics approval for ABRIDUS was granted by Curtin University Ethics Committee, University of NSW Ethics Committee and the Victorian Department of Human Services Ethics Committee. Full details of the methods used in ABRIDUS are described elsewhere [37].

### Participants and sampling

Participants were recruited in Melbourne (n = 150), Sydney (n = 150), and Perth (n = 150) and selected on the basis of having injected drugs at least monthly for the past six months. A targeted sampling strategy was used, whereby sample stratification was used to ensure that adequate numbers of females, IDU aged less than 25 years, and drug treatment naive participants were recruited (at least one third of each). Participants were recruited through Needle and Syringe Programs, drug and alcohol services, youth services, drug user groups, and snowball methods in each capital city.

### Weighting of BBV-TRAQ items

Item weights were assigned according to expert key informant ratings of HCV risk practices performed during the development stages of the BBV-TRAQ to help establish the construct validity of the scale [34]. Key informants were asked to rate items according to their relative risk of transmitting HCV on a scale between 0 (no risk) and 10 (highest risk). Median item ratings were then categorised into five groups used to assign item weights according to relative HCV transmission risk (see Table 1).

**Table 1:** BBV-TRAQ item categorisations and median key informant ratings

Item	Description	Median Rating
Category 5 (highest risk; median KE ratings 9–10)		

a5	In the last month, how many times have you injected a drug which has been filtered through another persons filter	9
a7	In the last month, how many times have you injected a drug prepared with water that had been used by another person	9
a8	In the last month, how many times have you injected a drug which had come into contact with another person's used needle and syringe	9
a13a	In the last month how many times have you injected with another person's used needle and syringe *a13b On those occasions, how often did you rinse it with a combination of full-strength bleach and water (2 × 2) before you used it*	10
a14	In the last month how many times have you injected with a needle and syringe after another person has already injected some of its contents.	10
a19	In the last month, how many times have you received an accidental needle-stick from another person's used needle and syringe	9
a20a	In the last month, how many times have you reused a needle and syringe taken out of a shared sharps container *a20b On those occasions, how often did you rinse it with a combination of full-strength bleach before you re-used it*	10
c2	In the last month, how many times have you been tattooed by someone who was not a professional tattooist	9
c3	In the last month, how many times have you been pierced by someone who was not a professional piercer	10

Category 4 (median KE ratings 7–8)		

a6a	In the last month, how many times have you injected a drug that was prepared in another persons used spoon or mixing container *a6b On those occasions how often did you clean the spoon or mixing container before using it*	7
a9a	In the last month, how many times have you injected a drug prepared immediately after assisting another person with their injection (injecting them, holding their arm, handling used fits, wipe blood away etc) *a9b On those occasions, how often did you wash your hands before preparing your mix*	8
a10a	In the last month, how many times have you injected a drug that was prepared by another person who had already injected or assisted someone else to inject *a10b On those occasions, how often did the person wash your hands before preparing your mix*	7
a11a	In the last month, how many times have you been injected by another person who had already injected or assisted in someone else's injection *a11b On those occasions, how often did the person injecting you wash your hands before injecting you*	Not rated
a12a	In the last month, how many times have you been injected with a needle and syringe which had been handled or touched by another person who had already injected *a12b On those occasions, how often did they wash their hands prior to handling the needle and syringe that you used*	7
a15a	In the last month, how many times have you touched your own injection site soon after 'assisting' another person with their injection *a15b On those occasions, how often did you wash your hands before touching your injection site*	8
a16a	In the last month, how many times has another person touched your injection site *a16b On those occasions, how often did they wash their hands before touching your injection site*	8
a17	In the last month, how many times have you wiped your own injection site with an object (swab, tissue, hanky) which had been used by another person	8
c4	In the last month, how many times have you used another person's used razor	7

Category 3 (median KE ratings 5–6)		

a1	In the last month, how many times handled another persons syringe at a time when you had cuts etc	6
a18	In the last month, how many times have you used a tourniquet which had been used by another person	6
b2	In the last month, how many times have you engaged in unprotected vaginal sex with another person *during menstruation*	5
c1	In the last month, how many times have you come in contact with another person's blood (fights, slash-ups, accidents, blood nose etc)	5

Category 2 (median KE ratings 3–4)		

a2	In the last month, how many times have you sucked or licked left-over drugs from spoon, mixing container used by another person	3
a3	In the last month, how many times have you sucked or licked a filter used by another person	3
a4	In the last month, how many times have you sucked or licked a plunger after using it in a mix used by another person	2.5
b3	In the last month, how many times have you engaged in unprotected vaginal sex with another person *without lubrication*	3
b4	In the last month, how many times have you engaged in unprotected *anal *sex with another person	4
c5	In the last month, how many times have you used another person's toothbrush	4
c6	In the last month, how many times have you used another person's personal hygiene equipment (nail file, nail scissors, brush etc)	3

Category 1 (lowest risk; median KE ratings 1–2)		

b1	In the last month, how many times have you engaged in unprotected vaginal sex with another person	1
b5	In the last month, how many times have you engaged in unprotected *oral *sex with another person	1
b6	In the last month, how many times have you engaged in unprotected *manual *sex with another person *during menstruation*	2
b7	In the last month, how many times have you engaged in unprotected *manual *sex with another person *after injecting*	2
b8	In the last month, how many times have you engaged in unprotected *manual *sex with another person *without lubrication*	1

There are evident challenges in attempting to apply empirical weights to scale items when there is a paucity of empirical data describing the relative risk associated with the specific practices described in those items. For example, what is the increased risk associated with, for example, sharing a needle and syringe over sharing other injecting equipment? To embark on a process of instrument refinement, an essential but habitually neglected process of improving a scale's representation of a construct [38], we examined the application of three weighting schemes applied to items within each of the five risk categories shown in Table 1. Log one (weights ranging from one to five for each risk category respectively); log two (weights of one, two, four, eight and 16 for each risk category respectively); and log five (weights of one, five, 25, 125 and 625 for each risk category respectively).

### Analysis

Detailed descriptions of the original BBV-TRAQ scoring system are reported elsewhere [34,39]. Only participants that answered all BBV-TRAQ questions were included. In this study, weights were applied to scores for each BBV-TRAQ item and then summed according to the factor structure derived from the original BBV-TRAQ development. Final weighted scores were obtained for injecting, other skin penetration, and sex subscales, and for the total scale [35]. Univariate descriptive statistics were generated for unweighted and weighted subscale and total scores.

Internal reliability was calculated for subscale and total scores using standardised item alpha. This internal consistency coefficient is preferred when individual items have different variances, with Cronbach's alpha being adversely affected by heterogeneity of item variances [40], and is appropriate here as individual items within subscales are weighted differently (thus producing marked differences in item variances). One-way ANOVA assessed differences in BBV-TRAQ scores according to the self-reported HCV status of IDUs ("HCV positive", "negative", "never tested", "don't know"). In an attempt to limit temporal biases of recent engagement in risk behaviours with HCV status, this between groups analysis was repeated across study sub-samples (i.e., time since first injected and age) to examine the stability of results among more recent initiates or less experienced injectors. Between groups analyses were also conducted on unweighted and weighted scores to explore the sensitivity of results to different weighting structures. Data were analysed using the SPSS Statistical Software Package Release 11.5.0.

## Results

### Sample profile

Of the 450 IDU surveyed in ABRIDUS, 419 (93%) completed all BBV-TRAQ items Melbourne (n = 142), Sydney (n = 133), and Perth (n = 144). Demographic and drug use characteristics of these participants are presented in Table 2. The sample was predominantly male and unemployed, most were heroin injectors, injected daily or more often, and had first injected drugs more than three years earlier. Nearly half (45%) the sample reported previously testing positive for hepatitis C, 14% reported previously testing positive for hepatitis B, and reported HIV prevalence was very low (2%). There were no systematic differences in the characteristics of those that completed all BBV-TRAQ items and those that did not.

**Table 2:** Demographic and drug use characteristics of IDU sample (N = 419)^1^

Percentage male			57% (239)
Mean age (SD)			27.8 (8.2)

Employment			
employed			22% (93)
student			6% (27)
unemployed			66% (275)
home duties			6% (24)

Mean years since first injection (SD)			9.5 (7.5)
≤ 3 years			23% (97)
> 3 years			77% (318)

Drug most injected in the past month			
heroin			71% (296)
amphetamines			22% (91)
other			7% (29)

Frequency of injecting in the past month			
weekly or less			20% (85)
more than weekly less than daily			26% (110)
daily or more			54% (224)

Percentage with no Tx experience			28%

Self-report BBV status	HCV	HBV^2^	HIV
positive	45% (189)	14% (60)	2% (7)
negative	42% (174)	38% (158)	87% (364)
never tested/not sure	13% (55)	16% (67)	11% (47)

^1 ^Some totals do not sum to 419 due to a small number of non-responders.

^2 ^Includes 32% (133) reporting previous HBV vaccination.

Table 3 presents sample descriptive statistics comparing original and weighted BBV-TRAQ scores. Both the weighted and unweighted scales produced positively skewed data with modes for total and subscale scores of zero (participants reporting no risk behaviours).

**Table 3:** Descriptive statistics for original and weighted BBV-TRAQ scores

**Score**	**Mean**	**Median**	**Standard Error**	**Standard Deviation**	**Minimum (number of cases)**	**Maximum**
Original BBV-TRAQ Scores						

Total Score	26	19	1.13	25.66	0 (31)	135
Injecting	15	9	0.79	16.13	0 (75)	87
Sex	8	2	0.47	9.62	0 (177)	40
Other Skin Penetration	3	2	0.18	3. 60	0 (155)	20

Log 1 Weighted BBV-TRAQ Scores						

Total Score	77	52	3.63	74.21	0	405
Injecting	59	36	3.12	63.84	0	332
Sex	10	3	0.63	12.83	0	60
Other Skin Penetration	8	5	0.48	9.84	0	55

Log 2 Weighted BBV-TRAQ Scores						

Total Score	160	105	8.08	165.31	0	805
Injecting	138	82	7.50	153.41	0	770
Sex	10	3	0.66	13.51	0	65
Other Skin Penetration	12	6	0.75	15.39	0	80

Log 5 Weighted BBV-TRAQ Scores						

Total Score	3,899	2,387	222.17	4,547.68	0	26,095
Injecting	3,745	2,325	217.71	4,456.33	0	25,970
Sex	21	3	1.79	36.72	0	200
Other Skin Penetration	133	25	14.44	295.61	0	2,500

### Reliability of the weighted BBV-TRAQ

Standardised item alphas were consistent across weighting schemes, showing good internal reliability for the total BBV-TRAQ (α = .89) injecting (α = .87) and sex (α = .89) sub-scales. A lower alpha coefficient was obtained for the other skin penetration (α = .49) subscale.

### Validity of the weighted BBV-TRAQ

Key expert rankings of items used in the original BBV-TRAQ established the construct validity of the scale [35]. Total and subscale weighted and unweighted BBV-TRAQ scores were compared between groups of participants according to their self-reported HCV status to explore potential predictive validity properties of the scale (see Table 4). Injecting subscale scores for log two and log five weights and total scores for log five weights were able to discriminate between HCV positive and negative IDU in the expected direction. Effects (as determined by specific *F *scores) increased with increasing item weights. Across all weighting schemes sex and other skin penetration scores were not significantly different across self-report HCV groups.

**Table 4:** Comparisons between self-report HCV status groups for unweighted and weighted BBV-TRAQ total and injecting subscales scores

Weights	BBV-TRAQ Score	F	p-value	mean difference (HCV positive & negative)	95% CI of the difference
None	Injecting subscale	2.46	.141	3	-1 – 7
	Total	0.76	.997	2	-4 – 8
Log one	Injecting subscale	3.04	.080	15	-1 – 31
	Total	1.98	.250	14	-5 – 32
Log two	Injecting subscale	3.63	.038	40	2 – 79
	Total	2.97	.078	39	-3 – 80
Log five	Injecting subscale	4.12	.017	1,293	176 – 2,411
	Total	3.86	.023	1,273	132 – 2,414

* Comparisons were made between all HCV groups (i.e., inclusive of 'never tested/not sure' group). To simplify tables only results relevant to the focus of this paper have been presented (i.e., 'HCV positive' and 'HCV negative'). No differences were detected between the 'never tested/not sure' and other groups.

Characteristics of the IDU sample (i.e., mean age, mean years since first injected; see Table 2) are indicative of a sample of well-established IDU who may have modified their risk practices over time. As such, it is potentially problematic to ask such older and more experienced IDU about their recent risk behaviours to explore potential predictive validity properties of the BBV-TRAQ. In order to account for and moderate the potential effects of a lengthy injecting career on the relationship between recent risk practices and HCV status, between groups analyses were repeated among more recent initiates to injecting (≤ 3 years; n = 96) and younger participants (≤ 25 years; n = 186). When compared to results from the whole sample (Table 4), both unweighted and weighted BBV-TRAQ injecting and total scores more clearly differentiated HCV positive versus negative IDU among younger aged participants and those that had initiated injecting recently. Consistent with results from the whole sample, the general trend was for increasing effects with increasing item weights (Table 5).

**Table 5:** Comparisons* between self-report HCV status groups and weighted and unweighted BBV-TRAQ total and injecting subscales scores among sub-samples of IDU

Sub-sample	Weight	BBV-TRAQ Score	F	p-value	mean difference (HCV positive & negative)	95% CI of the difference
Initiated injecting ≤ 3 years	None	Injecting subscale	3.93	.033	10.76	0.63 – 20.88
		Total	2.65	.076	14.5	-1.05 – 30.05
	Log 1	Injecting subscale	4.48	.016	46.35	6.66 – 86.04
		Total	4.19	.018	54.35	7.12 – 101.58
	Log 2	Injecting subscale	5.49	.005	120.02	28.99 – 211.04
		Total	5.41	.006	130.32	31.25 – 229.39
	Log 5	Injecting subscale	7.11	.001	3,696	1,278 – 6,113
		Total	7.19	.001	3,752	1,298 – 6,205

Aged ≤ 25 years	None	Injecting subscale	12.50	<.001	12.97	6.69 – 19.24
		Total	8.97	<.001	17.15	7.36 – 26.93
	Log 1	Injecting subscale	13.66	<.001	53.88	28.95 – 78.82
		Total	12.08	<.001	61.28	31.15 – 91.41
	Log 2	Injecting subscale	14.46	<.001	134.11	73.86 – 194.36
		Total	13.70	<.001	143.11	77.05 – 209.16
	Log 5	Injecting subscale	14.16	<.001	3,915	2,132 – 5,698
		Total	13.69	<.001	3,939	2,114 – 5,764

* Comparisons were made between all HCV groups (i.e., inclusive of 'never tested/not sure' group). To simplify tables only results relevant to the focus of this paper have been presented (i.e., 'HCV positive' and 'HCV negative'). No differences were detected between the 'never tested/not sure' and other groups.

## Discussion

By retrospectively examining original scale development and subsequent cross-sectional data, we have devised a new risk behaviour weighting system that further refines the BBV-TRAQ instrument and enhances the utility of the scale for HCV surveillance and prevention evaluation purposes. The approach reported here is consistent with classical scale development methods [38,41,42]. Both normative subscale scores and between group (HCV status) differences can be used to recommend the choice of a BBV-TRAQ weighting scheme to measure HCV transmission risk among IDU.

First, we can examine the contribution of subscales to total BBV-TRAQ scores in light of the plausible contributions injecting, sex and other skin penetration behaviours have to overall risk of HCV infection. The descriptive statistics presented in Table 3 show average subscale scores for the weighted and unweighted BBV-TRAQ. In terms of rank order contribution, both unweighted and log one-weighted scores can be discounted on the basis of emphasising sexual risk practices over other skin penetration practices, contrary to current opinion [22,25].

Examining the magnitude of subscale contributions, the sample data presented here shows that the log five-weighted scale is the most plausible method of modelling a relative risk profile using the BBV-TRAQ. It is well established that injecting risk behaviours (e.g. re-use of used needles and syringes) represent the greatest contribution to HCV incidence among IDU. In addition, recent research suggests 'extremely low or even null' risk of sexual transmission of HCV [30,31]. A log five weighting scheme that proposes injecting behaviours contributing in excess of 95% of overall HCV risk among IDU and sexual behaviours contributing less than one percent therefore offers a credible overall risk profile.

Estimating the role of other skin penetration practices on HCV transmission is less clear, with studies showing large variation in odds ratios attributable to tattooing [26], and the confounding factors of IDU and unprofessional tattooing, particularly in prison [23,25]. However, given the low or no risk of HCV sexual transmission, the blood-to-blood nature of HCV transmission and available epidemiological evidence, the larger relative risk contribution of other skin penetration versus sexual risk practices for the log five weighted scale again appears more realistic.

Second, examining differences in scores between self-report HCV positive and negative participants also lends credence to the log five-weighted scale. The log-five weighted total and injecting sub-scale scores were able to discriminate between HCV positive and negative IDU across the entire sample and among sub-groups of younger and less experienced users. Although requiring prospective sero-incident data to reliably establish predictive validity among IDU, the cross-sectional data reported here show log five weighted BBV-TRAQ scores able to differentiate levels of risk between HCV positive and negative participants in the expected direction. These results show that the application of item weights further refines the BBV-TRAQ instrument by presenting a more precise measure of individual risk of contracting HCV across a broad range of risk behaviours.

### Study strengths and weaknesses in relation to other studies

Other studies investigating injecting risk practices associated with HCV status have reported mixed results. Some have reported no differences in rates of sharing between HCV positive and negative IDU [43,44], suggesting that the targeting of individual risk behaviours may have little utility. Others [10,45-47] have demonstrated differences in self-reported injecting risk behaviours according to the HCV status of IDU.

One possible explanation for the discrepancies in results is the format of risk behaviour questions asked of IDU. Those reporting no differences have reported 'catch-all' responses about the sharing of 'needles and syringes' and the sharing of 'other equipment' with dichotomised yes/no response categories. Assuming participants were asked about their risk behaviours in the same way their responses were reported and analysed, such measurement may under-estimate the sharing of injecting equipment. Indeed, others have found the reported prevalence of injecting risk practices increases markedly when more detailed questions about the sharing of specific injecting equipment are asked [21,48].

Graded response options, as used in the BBV-TRAQ, may help to limit recall bias and aid in the collection of more accurate injecting risk practice data [19]. This may be particularly valid among highly socialised injectors where 'accidental or unnoticed sharing of equipment' might contribute to HCV transmission risk [49-52]. It is also possible that multiple domain graded-response instruments such as the BBV-TRAQ might be more likely to trigger the memory of participants' risky injecting behaviours, reduce recall bias, and subsequently enhance the likelihood of detecting associations between injecting behaviours and HCV status. In some studies, graded frequency scales have been employed with success in multiple questions about the sharing of individual items used in the preparation and injecting process [10,45-47].

### Implications for clinicians and policymakers

One of the key advantages of having a standardised HCV risk practice assessment instrument with a weighted scoring system is that it can be applied widely as a measure of the likelihood of HCV infection. Further validation using prospective seroincident data could see such a scale used as a reliable screening tool and as a surrogate indicator of seroconversion, particularly among IDU, providing a low cost alternative to serology testing.

For example, the BBV-TRAQ could potentially be adopted as an outcome measure to evaluate community-based HCV prevention activities, particularly those that target high-risk behaviours identified in the BBV-TRAQ, and inform the development of future education and prevention initiatives in specific settings. Because the scale is brief, easy to administer and score, and inexpensive to apply [34,35], adopting the BBV-TRAQ as an outcome measure overcomes some of the impediments inherent in evaluating the efficacy of interventions to reduce HCV transmission. This can be particularly valuable in circumstances where resources, time, and the capacity to recruit and follow large numbers of prevention trial participants is limited, as can often be the case in community-based or non-government organisation prevention settings.

In addition, the instrument offers opportunities for empirically scaled standardised population surveillance of HCV risk behaviour that go beyond recording dichotomised responses indicating if someone had recently engaged in a risk practice or not. By covering the full range of injecting and putative HCV risk practices in combination with protective practices, the BBV-TRAQ also offers education opportunities to IDU by highlighting risk behaviours and opportunities for risk reduction. Our experience using the instrument in standard descriptive epidemiological studies [37] and recent brief intervention research [53] indicates that the scale has some impact for people completing it by accentuating both risk and risk reduction behaviours. Using BBV-TRAQ normative data, respondents could be categorised into BBV risk categories with prevention education tailored accordingly, and could also be provided with qualitative feedback that highlights particular risk behaviours that contributed most to their overall BBV-TRAQ score.

Needless to say the ability for individuals to adopt risk reduction behaviours is affected by numerous social and structural factors [15,43,54-56]. Whereas answering questions about *why *people adopt or maintain practices that place them at risk of HCV infection are best explored using ethnographic and qualitative methods [15], the BBV-TRAQ would ideally sit alongside such methodologies by describing *what *practices people actually engage in and the *frequency *with which they occur.

### Strengths and weaknesses of the study

A number of methodological limitations need to be considered when interpreting these results. The most salient limitation is the reliance upon self-reports to characterise the HCV status of IDU. Researchers assessing self-report HCV and HBV status among IDU have reported variable validity [57,58], however, there is evidence that questionnaire self-administration (the BBV-TRAQ is a self-administered instrument) increases the reliability of self-report of behaviours relating to sensitive topics [59]. A recent study reported that 72% of HCV positive IDU and 46% of HCV negative IDU were not aware of their HCV status [60]. This study was, however, conducted across five US cities and found that use of drug treatment or needle and syringe programs was associated with increased awareness of HCV status. Given the more comprehensive nature of Australia's long-established needle and syringe program network and a well recognised harm reduction framework with regards to injecting drugs, one might expect that knowledge of HCV status among Australian IDU would be higher than those reported in the US. In addition, the rates of HCV infection in our sample (45%) are similar to other comparable Australian samples in which serological data were collected (e.g., 50%; [6]). However, it is the case that some anti-HCV positive IDU may have cleared the virus, whereas others may have mistakenly identified themselves as HCV positive or negative. The latter misclassification is the most salient here, because associations between BBV-TRAQ scores and HCV may in fact reflect an underlying association with awareness of HCV status. Previous research among Australian IDU showed an association between HCV testing and greater knowledge about BBV transmission and safe injecting [61]. Although such results would suggest that participants in this study that were tested for HCV more often or more recently might more reliably report their HCV status, we cannot assume that this reliability would be more biased towards self-report HCV positives or negatives. However, if we assume similar HCV knowledge characteristics in this sample as those reported by Treloar et al [61] and also take account of the HCV status awareness reported above in the US [60], then it is possible that self-report HCV negatives may be more likely to report safer injecting practices on the BBV-TRAQ.

A second limitation is the cross-sectional data collection and the temporal differences between reporting risk behaviours and infection. To definitively test the suitability of scale weights among IDU (and reliably establish predictive validity), the BBV-TRAQ would need to be examined within the context of prospective sero-incident studies. In most cases, however, changes in risk behaviour profiles are theoretically likely to favour reductions in risk over time as IDU are exposed to more harm reduction messages and more contacts with service providers [60,62]. Combined with cumulatively more opportunities for viral transmission over time, such temporal changes would be more likely to diminish the ability of recent risk behaviours to predict lifetime exposure to HCV. Indeed, the analyses conducted on study sub-samples of younger and less experienced injectors shows an enhanced effect for the weighted BBV-TRAQ to distinguish between self-report HCV positive and negative participants (see Table 5). Although not a substitution for a prospective sero-incident study, examining HCV prevalence among recent initiates to injecting drugs has previously been used to help with indirect estimates of HCV incidence [63].

Consistent with results from other studies [10,43], injecting and total weighted BBV-TRAQ risk scores in younger less experienced IDU were considerably higher (in the order of three times) than those for the whole sample combined. Results from prospective cohort work in the US with recent initiates to injecting drug use suggest that seroconversion could occur in a quarter of IDU within approximately two years of initiating injecting and in at least half within four years of initiation [10]. In addition, a recent Australian cohort study reported high HCV incidence among new initiates, with mean time to seroconversion of 1.6 years among participants injecting for less than two years [64]. In this context, results showing BBV-TRAQ scores better able to discriminate between HCV positive and negative recent initiates must give greater confidence (even if only as a surrogate measure) in the predictive validity of the weighted BBV-TRAQ. It is likely that recent injecting risk practices among less experienced injectors would be somewhat indicative of their lifetime injecting risk practices, strengthening the connection between self-reported recent behaviours and lifetime exposure to HCV.

Finally, the BBV-TRAQ has largely been developed using IDU cohorts. By testing this weighted instrument with IDU we are not assuming that this group is the only one at risk of HCV infection. IDU are, however, the predominant risk group in many countries [4-6] and therefore constitute the most relevant group from which to begin establishing the reliability and validity of the weighted BBV-TRAQ. As described below, testing the validity of the BBV-TRAQ with other populations is needed. For example, testing the suitability of the instrument for non-injection routes of transmission with people not currently injecting but considered at some level of risk according to BBV-TRAQ scores.

### Unanswered questions and future research

The psychometric development and refinement of a scale is a necessarily iterative process [38]. Of fundamental importance in early scale development stages is the establishment of construct and content validity [65,66], properties that were emphasised and assiduously developed during the initial development stages of the BBV-TRAQ [34,35]. Building on this foundation, this paper presents data on refinements to the BBV-TRAQ that strengthen its representation of the underlying construct of BBV risk assessment – in particular behavioural risk practices for HCV infection among IDU.

There are two important areas of future research warranted to enhance the properties and utility of the scale. First, in order to overcome the temporal limitations of relating lifetime infection with recent risk behaviours and reliably confirm the predictive validity of the scale, the BBV-TRAQ needs to be incorporated into prospective incidence studies of HCV transmission that involve the serial testing over time of baseline HCV-negative IDU. Second, as the properties of the BBV-TRAQ have largely been examined in the Australian IDU context with primary heroin injectors, subsequent studies are needed to explore these properties across different cultural, socio-environmental, disease prevalent and drug market conditions. How scores for the BBV-TRAQ might translate to other populations with different drug markets (e.g., cocaine injecting) or different harm reduction capacities (e.g., more restricted access to clean injecting equipment) is unknown. The utility of the instrument for determining risk of HIV and HBV infection among IDU and other at risk groups also needs to be examined in populations with higher disease prevalence. In this regard, no scale development occurs with all population groups at one time. As such, it is important to publish data on this stage of scale refinement as an iterative step in providing normative data, upon which data collection and analysis of results from different populations can be built.

## Conclusion

In this study we have refined the BBV-TRAQ to recommend the use of a log five weighted scoring system to quantify HCV transmission risk among IDU. The application of such weights resulted in credible relative risk scores across subscales and provided a clear distinction between HCV positive and negative IDU, particularly among younger and less experienced injectors, for both total and injecting subscale BBV-TRAQ scores. Reliable, valid and easy to administer, the BBV-TRAQ is ideal for use in community settings for research and surveillance purposes, and for the evaluation of BBV education and prevention initiatives.

## Competing interests

The authors declare that they have no competing interests.

## Authors' contributions

MS conceptualised the weighting scheme and the analytical approach to testing the properties of the weighted BBV-TRAQ. MS conducted the analyses presented in this paper, provided content knowledge and made major contributions to the written manuscript. CF was the principal researcher on the original development of the BBV-TRAQ and for the ABRIDUS. CF contributed content knowledge and made major contributions to the written manuscript. NL was a co-researcher on the original development of the BBV-TRAQ, provided content knowledge and clinical expertise to this paper and made contributions to the written manuscript. All authors read and approved the final manuscript.
